# Revealing gender differences in concussion reporting: a detailed analysis of SCAT assessment self-report symptom ratings

**DOI:** 10.3389/fneur.2025.1584875

**Published:** 2025-10-22

**Authors:** Rachel Edelstein, Karen M. Schmidt, John Darrell Van Horn

**Affiliations:** ^1^Department of Psychology, University of Virginia, Charlottesville, VA, United States; ^2^UVA School of Data Science, Charlottesville, VA, United States

**Keywords:** SCAT3, concussion, gender differences, symptom reporting, NCAA athletes, psychometrics

## Abstract

**Introduction:**

Current concussion assessments used by the NCAA are generally applied to both male and female athletes to evaluate the effects of sports-related head impacts. However, increasing evidence indicates that female athletes show different physiological and psychosocial responses to concussions compared to their male counterparts, raising concerns about the suitability of gender blind concussion assessments.

**Methods:**

This study analyzes data from *N* = 1,021 NCAA athletes (379 females, 642 males) who completed the SCAT3 Symptom Severity Checklist after a concussion. A systematic use of multivariate statistical methods, including Exploratory Graph Analysis (EGA), Principal Component Analysis (PCA), Exploratory Factor Analysis (EFA), Linear Discriminant Analysis (LDA), and Rasch Partial Credit Modeling (PCM), was applied to this 22-item instrument to explore the underlying factor structure and identify assessment items sensitive to gender differences. Differential Item Functioning (DIF) analysis examined gender disparities in symptom reporting.

**Results:**

Based on EGA and PCA, the SCAT3 showed a four-factor substructure, with EFA accounting for 62.44% of the variance. LDA comparing males and females revealed a significant difference in their multivariate score distributions (*χ*^2^ (22) = 130.56, *p* < 0.001), with emotional and physical symptom items loading negatively, and cognitive and sensory items loading positively. This suggests emotional and physical symptoms contribute oppositely to cognitive and sensory symptoms, implying these domains may represent opposite ends of a single symptom dimension. Rasch analysis of each assessment item identified three items with no difference between genders. Conversely, nine symptoms showed males were more likely to report higher severity. Nonetheless, females generally reported divergent overall symptom severity scores (Mean = 30.06, SD = 20.88) than males (Mean = 24.71, SD = 21.18), t(765.06) = 3.85, *p* < 0.001.

**Discussion:**

Differences in symptom presentation post-concussion may suggest that: (1) males tend to be more conservative in reporting and only endorse symptoms when they are more intense, leading to higher scores on fewer symptoms, whereas (2) females may more readily emphasize emotional and physical symptoms. The findings imply that considering gender differences in concussion symptom reporting is important when making clinical recommendations.

## Introduction

Sports-related concussions (SRC) represent a significant health concern for student-athletes regardless of age, sport, athletic division/conference/etc., or gender. The *National Collegiate Athletic Association (NCAA)* employs a broad, multifaceted approach to determining SRC, but one which relies heavily upon symptom self-reporting as a primary method of assessment (1–6). However, increasing evidence suggests that male and female athletes exhibit distinct physiological and psychosocial responses to concussions, raising concerns about the adequacy of the current gender-neutral diagnostic framework (7–14). Despite this growing body of research, NCAA concussion assessments, such as the widely used *Sport Concussion Assessment Tool (SCAT)*, do not specifically account for gender differences in symptom reporting or recovery trajectories (15).

Concussion rates among NCAA student-athletes underscore the importance of refining diagnostic tools. Over 460,000 student-athletes participate in NCAA competitions each year, nearly half (43.7%) of whom are female (16, 58). For instance, between the 2009–2010 and 2013–2014 academic years, approximately 4.47 concussions occurred per 10,000 athlete exposures, resulting in around 10,560 concussions annually (17, 18). Women’s soccer ranked second among sports with the highest concussion rates (19). In spite of differences in concussion exposure, NCAA concussion assessment protocols have tended to remain gender-agnostic, applying the same self-report diagnostic tools to male and female athletes alike (4, 6, 20).

Research consistently shows that male and female athletes differ in both the frequency and severity of reported concussion symptoms. Studies have indicated that female athletes tend to report a higher frequency of concussions, along with more severe symptoms, compared to their male counterparts (7–9, 21, 59, 60). These gender differences suggest that the current gender-neutral assessment methods may overlook key factors influencing symptom expression and recovery outcomes, potentially compromising the accuracy of concussion management (22–24). Furthermore, a systematic review of gender differences in SRC revealed that female athletes often experience longer recovery times, though the exact reasons for these differences remain uncertain (25). Such discrepancies may stem from biases in the diagnostic tools, which have historically been developed using predominantly male populations (26, 27).

### The Sports Concussion Assessment Tool (SCAT)

The *Sports Concussion Assessment Tool (SCAT)* is a widely used assessment measure developed to systematically evaluate symptoms and cognitive functioning in athletes who may have experienced a concussion (61, 62). Initially introduced in 2004, following *the 2nd International Symposium on Concussion in Sport*, organized held in Prague, Czech Republic, and subsequently updated (SCAT2 in 2008, SCAT3 in 2013, SCAT5 in 2017, and SCAT6 in 2023; The version number SCAT version 5 was chosen to align the version number with the *5th International Consensus Conference on Concussion in Sport*, held in Berlin, Germany, in 2016, meeting number and, as such, there is no SCAT4), the SCAT reflects advancements in concussion research and clinical feedback aimed at standardizing concussion assessment and improving its sensitivity. A depiction of the historical development of the SCAT components is presented in Table 1.

**Table 1:** Versions of the Sports Concussion Assessment Tool (SCAT).

Original SCAT	SCAT2	SCAT3	SCAT5	SCAT6
2004	2008	2013	2017	2023
*A: 22-Item Symptom Evaluation*	*22-Item Symptom Evaluation*	*Potential Signs of Concussion? (e.g., “Any loss of consciousness?”)*	*1: Step 1: Red Flags*	*Athlete Information and Concussion History*
B: Medical Evaluation 1—Signs 2—Memory 3—Symptom score 4—Cognitive assessment 5—Neurological screening 6—Return to play	1: “Symptom Score” (22 minus the number of reported symptoms)	1: Glasgow Coma Scale (GCS)	1: Step 2: Observable Signs	Step 1: Observable Signs (Witnessed or Observed on Video)
	2: Physical Signs Score	2: Maddocks Score	1: Step 3: Memory Assessment – Maddocks Questions	Step 2: Glasgow Coma Scale (GCS)
	3: Glasgow Coma Scale (GCS)	3: How do you feel? (*22-Item Symptom Evaluation*)	1: Step 4: Examination/Glasgow Coma Scale (GCS)	Box 1: Red Flags
	4: Sideline Assessment – Maddocks Score	4: Cognitive assessment (Standardized Assessment of Concussion (SAC))	1: Cervical Spine Assessment (three items)	Step 3: Cervical Spine Assessment
	5: Cognitive Assessment (Standardized Assessment of Concussion (SAC))	5: Neck Examination (range of motion/tenderness/limb sensation)	2: Step 1: Athlete Background (including past concussion history)	Step 4: Coordination and Ocular/Motor Screen
	6: Balance Examination	6: Balance Examination	2: Step 2: Symptom Evaluation (*22-Item Symptom Evaluation*)	Step 5: Memory Assessment – Maddocks Questions
	7: Coordination Examination	7: Coordination Examination	3: Step 3: Cognitive Screening (Standardised Assessment of Concussion (SAC)) Orientation Immediate Memory Concentration: Digits Backwards Months in Reverse Order	Off-Field Assessment – Step 1: Athlete Background
	8: Cognitive Assessment	8: SAC Delayed Recall	4: Step 4: Neurological Screen	Step 2: Symptom Evaluation (*22-Item Symptom Evaluation*)
	Overall Score and Summary	Overall Score and Summary	5: Delayed Recall	Step 3: Cognitive Screening (based on the Standardized Assessment of Concussion (SAC)) Immediate Memory Concentration
			6: Step 6: Decision (Overall Score and Summary)	Step 4: Coordination and Balance Examination Modified Balance Testing (mBESS) Timed Tandem Gait Dual Task Gait (optional)
				Step 5: Delayed Recall
				Step 6: Decision (Overall Score and Summary)

While the 22-item self-report Symptom Evaluation component of the SCAT (shaded in white) has shifted its position in the list of assessments to be completed with each new version of the SCAT, the Symptom Evaluation component has remained as a consistent element of the instrument since its inception. As additional elements have been added to the SCAT, however, no sub-scaling of the Symptom Evaluation has implemented, nor has there been any inclusion of female-athlete specific components within the SCAT.

The SCAT incorporates both subjective symptom self-reporting and objective cognitive and physical assessments. The symptom evaluation component, which lists common concussion symptoms such as headaches, dizziness, nausea, and mental fog, requires athletes to self-rate the severity of each symptom. This self-reporting is meant to align with the recognition that subjective symptomology is an essential indicator of concussion severity and recovery. Alongside symptom evaluation, SCAT includes a cognitive assessment that tests immediate and delayed memory, concentration, and orientation, as well as balance testing through the modified Balance Error Scoring System (BESS). SCAT has been widely adopted in sports, particularly at professional and amateur levels, and is endorsed by major organizations such as the *International Olympic Committee (IOC)* and *World Rugby*.

The SCAT5 introduced several refinements over the SCAT3 to enhance usability and clinical accuracy. One notable change was the expansion of the symptom checklist to accommodate a broader range of neurological symptoms and increased sensitivity to symptom severity. This update was performed in response to research on the diverse ways concussions present, aiming to capture subtle changes that could be missed in earlier iterations. SCAT5 also offers updated guidance on interpreting symptom severity scores, including clearer thresholds to guide clinical decisions about when athletes should return to play (28–30, 63). Additionally, it emphasizes cognitive and neurological examination components, enhancing sections on memory, concentration, and balance testing. For instance, the SCAT5 includes more detailed instructions for assessing balance using the modified Balance Error Scoring System (BESS), an essential metric for detecting vestibular and motor impairments post-concussion. Moreover, the SCAT5 provided updated guidelines on its utility across age groups, particularly recommending modifications for athletes under 13 with the Child SCAT5. Overall, changes in SCAT5 sought to provide a more robust framework, allowing clinicians to identify and manage concussions with greater confidence and precision compared to the SCAT3. The current SCAT6 version (released in 2023), extends the set of neurological assessment domains still further (see below as well as in Table 1).

However, the self-report symptom rating portion of the SCAT has remained a consistent core feature across all iterations, from its initial version in 2004 to the SCAT6, despite numerous enhancements to other sections of the tool (15). This feature in the assessment reflects the central influence of subjective symptom reporting in concussion diagnosis and management, as athletes’ descriptions of their symptoms provide critical insights that objective or more clinical observation measures may not fully capture.

In the self-report portion of the SCAT, athletes rate the severity of various concussion symptoms—such as headache, nausea, dizziness, and cognitive fog—on a scale from 0 (none) to 6 (severe), creating a total symptom score. This scoring approach has not changed, even as additional assessment components, like more detailed cognitive testing, neurological assessment, and balance evaluations, have been added to enrich the SCAT’s comprehensiveness. Notably, throughout the history of the SCAT, there has never been any particular differentiation between the experience of female versus male athletes in response to the perceived symptoms of concussion and the assessment provides no sub-scaling nor differentiation between male and female symptom reporting (see Table 2).

**Table 2:** Demographics.

	Females	Males	Total
*N*	379	642	1,021
Age	19.76 (1.22)	19.92 (1.47)	19.87 (1.34)
Basketball	83	58	141
American football	0	415	415
Lacrosse	46	43	89
Soccer	121	54	175
Softball	50	0	50
Volleyball	79	0	79
Water Polo	0	40	40
Wrestling	0	32	32

Mean (SD). All participants participated in contact or collision sports, such as football, soccer, basketball, lacrosse, softball, volleyball, water polo, and wrestling.

### The NCAA-DoD concussion assessment, research, and education (CARE) consortium

The NCAA-DoD *Concussion Assessment, Research, and Education (CARE) Consortium* represents a large-scale, multi-institutional research initiative focused on understanding SRC, primarily in college athletes and military cadets. Launched in 2014 as a collaboration between the NCAA and the U.S. Department of Defense (DoD), CARE aims to advance knowledge on concussion diagnosis, management, and recovery, as well as long-term health outcomes (31). This consortium integrates data from baseline assessments, injury evaluations, and post-injury follow-ups to study concussion trajectories comprehensively. Involving over 30,000 athletes across 30 + institutions, CARE represents one of the largest databases of concussion data globally. Its assessments encompass a wide range of modalities, including self-reported symptoms, neurocognitive testing, neuroimaging, and genetic analyses, allowing for a multidimensional understanding of concussion’s impact on brain health. The CARE Consortium collects pre-injury baseline data from athletes, enabling comparisons between pre-and post-concussion states and for tracking recovery progress in detail (31). This data-design has allowed for insights into gender, age, and sport-related differences in concussion risk and recovery, as well as the development of sophisticated predictive models for post-concussive outcomes. Through its broad dataset, CARE supports numerous research initiatives to develop evidence-based clinical guidelines, improve safety protocols, and identify biomarkers that could lead to more accurate diagnosis and personalized treatment for concussions, with impacts extending beyond sports medicine to military and civilian healthcare.

All data from the CARE Consortium have been made openly available on the *Federal Inter-Agency Traumatic Brain Injury Resource (FITBIR)* data archive (fitbir.nih.gov). This includes clinical assessments, neuroimaging, balance test metrics, etc. The version of the SCAT used by the CARE Consortium for which data is available in the FITBIR archive was obtained using the SCAT3 version of the assessment, so while not reflective of the current state-of-the-art concerning the SCAT, it reflects the same core set of 22 self-report items as the more recent, SCAT6, assessment.

### Scoring the SCAT self-report assessment

The SCAT6 incorporates several clinical and cognitive test components designed to provide a comprehensive assessment of concussion effects on an athlete. The clinical component begins with an “Immediate or On-Field Assessment” to identify any severe injury markers, termed “Red Flags,” such as neck pain or altered consciousness, which necessitate urgent medical attention. This is followed by an orientation and memory section, in which athletes answer basic questions regarding time, place, and recent events, aiding in the identification of disorientation or memory loss. The cognitive portion includes immediate memory recall, where athletes repeat a list of words presented to them, testing short-term memory, and a concentration test involving number sequencing and reverse recitation, which assesses focus and mental processing. Additionally, the delayed recall component tests retention by asking athletes to recall the initial list of words after a brief delay, providing insight into memory consistency over time. The SCAT6 also integrates a modified Balance Error Scoring System (BESS), which assesses postural stability by having the athlete balance in various stances while clinicians score any errors in posture or movement. Together, these tests evaluate an athlete’s cognitive functioning, memory, and balance—key areas frequently affected by concussion—helping clinicians make informed decisions about diagnosis, treatment, and readiness for return to play. Specifically, athletes complete a cognitive screening that includes orientation questions, immediate memory recall, concentration tasks (like “serial 7 s” or months-in-reverse-order), and delayed recall; each scored separately. Physical testing, such as the modified Balance Error Scoring System (BESS), evaluates the athlete’s postural stability by measuring errors made during various stances.

Total scores on the SCAT do not yield a simple “pass/fail” outcome; instead, high scores indicate a generally more significant symptom burden and/or level of impairment. While no universal threshold score dictates whether an athlete is concussed, clinicians compare SCAT scores against baseline scores, if available, to detect changes and monitor recovery. However, the underlying basis of all versions of the SCAT checklist is the assumption that symptom severity can be captured by the *Total Symptom Score*, regardless of gender (32, 33). This overall number is then frequently employed to make clinical determinations on an athlete’s concussion severity and, ultimately, any clinical response. However, research by the CARE Consortium has suggested that the pooling of responses from across the range of SCAT items may not reflect the more subtle elements of head injuries in both male and female athletes (31, 34).

### Considering the definition of gender

The interchangeability of the terms “gender” and “sex” in SRC research further complicates the issue of considering the differential effects concerning a spectrum of gender identities. “Sex” refers to biological differences between males and females, while “gender” encompasses the social roles, behaviors, and identities associated with each (64). Sports are commonly classified as “men’s” or “women’s” versions, based upon the biological interpretation. This distinction is nuanced but essential for understanding how social and cultural factors may influence symptom reporting and recovery. The SCAT also makes no attempt to capture differences which may be relevant to transgendered athletes. Beyond these issues, most modern concussion assessment tools, including the SCAT checklist, would ideally need to adequately consider how factors related to the gender of the athlete could skew symptom evaluation and recovery outcomes. In a broader sense, the consideration of SRC across the spectrum of perceived gender identities is beyond the scope, per se, of the present investigation, and the consideration of gender is limited to male and female labels, as reported by the CARE Consortium in the FITBIR archive.

### Examining the structure of the SCAT and the potential for gender differences

Under the SCAT assessment, female athletes are more likely to report a broader range of symptoms with greater severity than males (35, 65). Thus, these findings highlight the need to reexamine the psychometric properties of the SCAT checklist to ensure it accurately reflects the symptomatology of both genders (27).

To address these gaps, the present investigation seeks to deconstruct the multidimensional nature of SRC symptom reporting, focusing on how gender may influence the perception and reporting of symptoms. By drawing on a robust dataset from the NCAA and DoD CARE Consortium, the study applies a suite of advanced multivariate statistical methods to assess the underlying dimensionality of concussion symptoms and to unravel the complexity of symptom reporting across genders. Exploratory Graph Analysis (EGA), Principal Component Analysis (PCA), Exploratory Factor Analysis (EFA), and Linear Discriminant Analysis (LDA) are employed to explore and clarify the dimensional structures that underlie the symptom clusters reported on the SCAT checklist, providing insights into how different symptoms co-vary and whether specific patterns emerge across male or female athletes.

As a complement to these methods, Differential Item Functioning (DIF) analysis and Rasch Modeling are used to rigorously investigate whether the SCAT checklist might disproportionately represent symptom severity scores in both male and female athletes, even when adjusting for differing individual trait level differences between genders. This layered approach aims to go beyond simple univariate symptom reporting or intensity comparisons and seeks to identify whether any gender-specificity exists in the underlying assessments themselves. Importantly, this study seeks to identify distinct clusters of concussion symptoms that more accurately reflect gender differences, helping to present a more nuanced, multidimensional framework for concussion assessment.

Given the SCAT’s historical consistency and comprehensive coverage of subjective concussion symptoms, a multivariate analysis of its self-report items is both timely and highly relevant, especially in light of the tool’s lack of adjustments or thresholds that account for the athlete’s gender. Since the SCAT self-report section has remained largely unchanged across iterations, this stability offers a unique opportunity for researchers to analyze symptom reporting trends over time and across diverse populations. Despite research showing that gender differences may influence concussion symptomatology and recovery trajectories, the SCAT does not differentiate between scores or assessment criteria based on gender, potentially overlooking nuanced variations in symptom reporting between male and female athletes. Multivariate analysis could reveal patterns and dimensions within self-reported symptoms that vary by gender, identifying clusters or specific symptom profiles that might be more predictive of prolonged recovery in one group compared to the other. By examining the dimensionality of symptom reporting with statistical rigor, this approach could provide valuable insights that might improve individualized concussion management. Such an analysis could support the development of more tailored concussion guidelines, refining both diagnostic and recovery protocols to account for gender-related differences, ultimately enhancing the clinical utility of the SCAT for both male and female athletes.

## Methods

### Demographics

N = 1,021 NCAA student-athletes (379 females and 642 males) completed the SCAT Version 3.0 (SCAT3) Symptom Severity Checklist within 48 h post-concussion, which was obtained from the *Federal Interagency of Traumatic Brain Injury Research (FITBIR)* in collaboration with the NCAA and DoD CARE Consortium. As noted above, the checklist includes 22 symptoms, each assessed using a 7-point Likert scale ranging from 0-to-6. These symptoms are summed for a *Total Symptom Severity Score,* in which values may range from 0-to-132.

### Statistical approaches

A systematic approach was utilized to evaluate the SCAT concussion assessment instrument’s underlying dimensionality. First, an exploratory graph analysis (EGA) was performed to illustrate the SCAT’s potential underlying multivariate structure (36). This was followed by a Principal Component Analysis (PCA) (37), which formed the basis for a subsequent Exploratory Factor Analysis (EFA) (38), which was used to determine which assessment items load the most onto latent symptom constructs (39). A linear discriminant analysis (LDA) was also performed to determine the most discriminating SCAT items between males and females (66). Lastly, the Masters (40) Partial Credit Model (PCM) and (67) Differential Item Functioning (DIF) analysis were conducted to confirm LDA results and provide greater specificity to gender-related symptoms on the SCAT symptom checklist most sensitive to differences between male and female athletes. PCA, EFA, and LDA analysis were also conducted through R version 4.2.2 (68). In what follows, we describe the details involved in each of these steps:

### Exploratory graph analysis (EGA)

Utilizing R version 4.2.2 in conducting EGA, running the *EGAnet* package version 1.2.3 (36). These network-based models use nodes to represent random variables connected by edges, indicating the level of unique interaction between them rather than individuals in networks, aiding in determining the number of dimensions through cluster detection.

### Principle component analysis (PCA)

PCA was utilized for dimensionality reduction while preserving as much of the variability in the data as possible, deconstructing the item-wise correlation matrix, and transforming the original variables into a new set of linear combinations of the original variables. These new variables, called principal components (PCs), are orthogonal (independent of one another) and ordered, so the first few retain most of the variation in all the original variables. The eigenvalues associated with each PC were examined, and those greater than or exceeding unity (e.g., Kaiser’s Criterion) were taken as indicative of the SCAT assessment’s multivariate sub-space.

### Exploratory factor analysis (EFA)

Building from the PCA and assessing the number of putative factors, an EFA was done to determine the final number of factors and the subsequent standardized loadings for each assessment item, loading onto each factor. Three and four-factor models with varimax rotation were compared by analyzing fit indices, computing the 𝜒^2^ fit statistic, RMSEA, the Comparative Fit Index (CFI), and the Tucker-Lewis Index (TLI) (41).

To determine clusters of items and their corresponding factor, a cut-off score for the standardized factor loadings of 0.21 comes from the approach where the smallest acceptable absolute factor loading is determined as one over the square root of the number of items (69). This is consistent with (70) concerning psychometric validation for ensuring robust factor interpretation and dimensionality.

### Linear discriminant analysis (LDA)

LDA finds the best linear combination of assessment items, which maximally separates two or more distributions relative to within-distribution variability (39). Moreover, LDA is solving an eigenvector (*w*) problem to maximize group separation. To determine this separation, the coefficients and corresponding eigenvalues (*λ*) were used to determine directions along the axes, computing the identification of symptoms best separated by gender. The eigenvector coefficient equation comes from solving the eigenvalue equation (A−λI)w=0, where A is a square matrix of the between-class and within-class scatter matrices (42). The eigenvector coefficients are the elements of the eigenvector, and these coefficients can be found by solving the linear system derived from the matrix A (Trendafilov and Gallo, 2021). Furthermore, these coefficients define how the features combine to form the maximal gender separation between symptoms.

Finally, the statistical significance of this discriminant function was evaluated using Wilk’s Lambda (⋀) statistic and its approximate F-ratio test statistic. A low ⋀ value approaching 0 and a significant *p*-value indicates that the discriminant function explains a substantial portion of the variance between the groups (43).

### Rasch analysis

#### Rasch partial credit model (PCM)

The PCM, a model within the family of Rasch measurement theories, was employed to analyze the response data (44). This model is particularly suited for handling ordinal response categories typical of symptom severity scales, offering robust estimations of item difficulty parameters without assuming equal distances between each category. The PCM equation can be interpreted as follows: *r* is the current step, *x* is the current step, and *m* is the full set of categories. The numerator sums up to the current category, while the denominator is the sum of all the categories:


$$P_{\mathrm{ix}}(\theta )=\frac{\exp[\sum_{j=0}^{x}\theta -\delta_{\mathrm{ij}}]}{\sum_{r=0}^{\mathrm{mi}}[\exp\sum_{j=0}^{x}\theta -\delta_{\mathrm{ij}}]}$$

Delta parameters δ*ij* are specified per item; δ*ij* is the step difficulty or location where 2 categories intersect (category intersections). Item locations (*𝛽i’s*) are typically obtained by taking an average of all the deltas (δ*ij*), the points along the latent trait continuum at which the likelihood of endorsing successive response categories increased (45, 46). The estimated thresholds for each symptom were used to identify the levels at which respondents were likely to move between response categories. Symptoms with disordered thresholds were marked for further review.

Before applying the PCM, the assumptions of unidimensionality, local independence, and monotonicity were tested. Unidimensionality was assessed through Exploratory Factor Analysis (EFA), ensuring that all symptom items measured a single latent trait (46). PCM assumes that the item parameters (e.g., symptom difficulties and thresholds) are invariant across different genders. This assumption implies that the model should work equally well across genders. Lastly, evaluating the assumption of monotonicity ensures that as the underlying trait increases, the probability of endorsing higher response categories also increases (40, 47).

#### Differential item functioning (DIF)

DIF occurs when individuals from different groups (e.g., genders) respond differently to a symptom despite having similar underlying latent trait levels. In the context of concussion symptom reporting, DIF quantifies the extent to which male and female athletes may interpret or report symptoms differently. In such cases, one gender may be more likely to endorse a symptom at a higher severity level than the other, even though their actual level of concussion-related impairment may be the same (40, 48). While measurement invariance across groups and time is desirable, cases in which symptoms are endorsed more severely for one gender versus another indicate lack of support for it. Ensuring measurement invariance is essential to ensure that the assessment accurately reflects an equivalent probability of endorsement for items for all individuals, regardless of gender (46). The following equation further computes the overall DIF measure or the difficulty for each gender and corresponding item


$$\beta_{\mathrm{ig}}=P(X_{\mathrm{ni}}=k\theta_{n},\delta_{\mathrm{ig}},\tau_{\mathrm{ikg}}\;)=\frac{\exp(\sum_{m=0}^{k}(\theta_{n}-\delta_{\mathrm{ig}}-\tau_{\mathrm{img}}))}{\sum_{j=0}^{M}\exp(\sum_{m=0}^{k}(\theta_{n}-\delta_{\mathrm{ig}}-\tau_{\mathrm{img}}))}$$

The DIF equation for the PCM involves several key variables (40). To compute the DIF measure for the corresponding gender and item β_ig_, the θ_n_ takes the ability level of person *n*, while *δ_ig_* is the difficulty of the item within each gender, *g*. The τ_img_ are step thresholds that define the boundaries between response categories.

DIF contrast quantifies the difference in item difficulty between groups (e.g., genders) for a specific symptom. The size and direction of the contrast indicate whether and to what extent males and females tend to report a higher severity (49). The equation below shows how the DIF contrast was computed, which takes the difference between the item difficulty for the prechosen reference group (females) and the focal group (males). This contrast value helps identify DIF direction and assess whether different genders respond differently to the same item after controlling for trait level (48). This is found using the following Masters (40) equation


$$\beta_{\text{contrast}}=\beta_{\text{if}}-\beta_{\mathrm{im}.}$$

Where *i* = item, *f* = female, and *m* = male. A negative contrast value of *β_if_​ − β_im_*​ suggests that females tend to more frequently report the symptom as relatively more severe than males at the same level of concussion severity (67). In other words, for the same overall concussion effects, females are more likely to endorse higher ratings for that symptom than males. Contrasts in the opposite direction, or a positive contrast, suggest that males tend to more frequently report the symptoms as relatively more severe than females.

To further determine significant symptoms that display DIF, Mantel–Haenszel probability statistics were used to determine whether an item exhibits uniform DIF between two observed groups, that is, whether an item is more frequently endorsed by one gender relative to the other, considering the latent trait. To avoid alpha inflation and Type I errors stemming from multiple comparisons, Benjamini & Hochberg (B-H) *post-hoc* tests (50) were conducted, as testing item DIF for many items poses an increased risk of Type I errors due to multiple tests with *α* < 0.05. Therefore, B-H correction was appropriate to control the false discovery rate (FDR) associated with the multiple comparisons (51).

## Results

### Demographics

As seen in Table 3, this analysis revealed a statistically significant difference in the mean Total Symptom Severity Scores between females (M = 30.06, SD = 20.88) and males (M = 24.71, SD = 21.18), t(765.06) = 3.85, *p* < 0.001. All participants were enrolled based upon participation in contact or collision sports, including football, soccer, basketball, lacrosse, softball, volleyball, water polo and wrestling, to ensure a consistent level of concussion exposure risk across the sample. The structure and breakdown of each sport can be described elsewhere (31).

**Table 3:** Item descriptive statistics.

	Females		Males		Total	
	*n* = 379		*n* = 642		*N* = 1,021	
Symptom	M	SD	M	SD	M	SD
Headache	2.52	1.29	2.38	1.54	2.45	1.42
Pressure in head	2.55	1.60	1.98	1.54	2.27	1.57
Neck pain	1.25	1.58	1.02	1.46	1.14	1.52
Nausea/vomiting	0.88	1.30	0.66	1.21	0.77	1.26
Dizzy	1.44	1.49	1.23	1.46	1.34	1.48
Blurry vision	0.67	1.16	0.68	1.18	0.68	1.17
Balance problem	0.77	1.19	0.71	1.19	0.74	1.19
Sensitivity to light	1.60	1.58	1.30	1.55	1.45	1.57
Sensitivity to noise	1.26	1.51	0.88	1.30	1.07	1.41
Feel slowed down	2.00	1.68	1.71	1.63	1.86	1.66
Feel in a fog	1.71	1.67	1.62	1.65	1.67	1.66
Do not feel right	2.23	1.70	2.04	1.70	2.14	1.70
Difficulty concentrating	1.89	1.68	1.54	1.66	1.72	1.67
Difficulty remembering	0.94	1.41	0.91	1.35	0.93	1.38
Fatigue/low energy	2.05	1.78	1.58	1.70	1.82	1.74
Confusion	0.68	1.13	0.77	1.27	0.73	1.20
Drowsiness	1.70	1.72	1.24	1.55	1.47	1.64
Trouble falling asleep	0.82	1.46	0.71	1.42	0.77	1.44
More emotional	0.94	1.47	0.79	1.32	0.87	1.40
Irritable	0.79	1.32	0.66	1.26	0.73	1.29
Sadness	0.64	1.27	0.39	1.02	0.52	1.15
Anxious	0.53	1.06	0.48	1.09	0.51	1.08
Symptom severity score	30.06	20.88	24.71	21.18	27.39	21.03

Means (M) and standard deviations (SD) for each of the 22 SCAT self-reported concussion symptoms and the total Symptom Severity Score are presented separately for female (*n* = 379) and male (*n* = 642) athletes, as well as the combined total sample (*N* = 1,021).

### Dimensionality of the SCAT self-report items

#### Exploratory graph analysis

**Figure 1:**
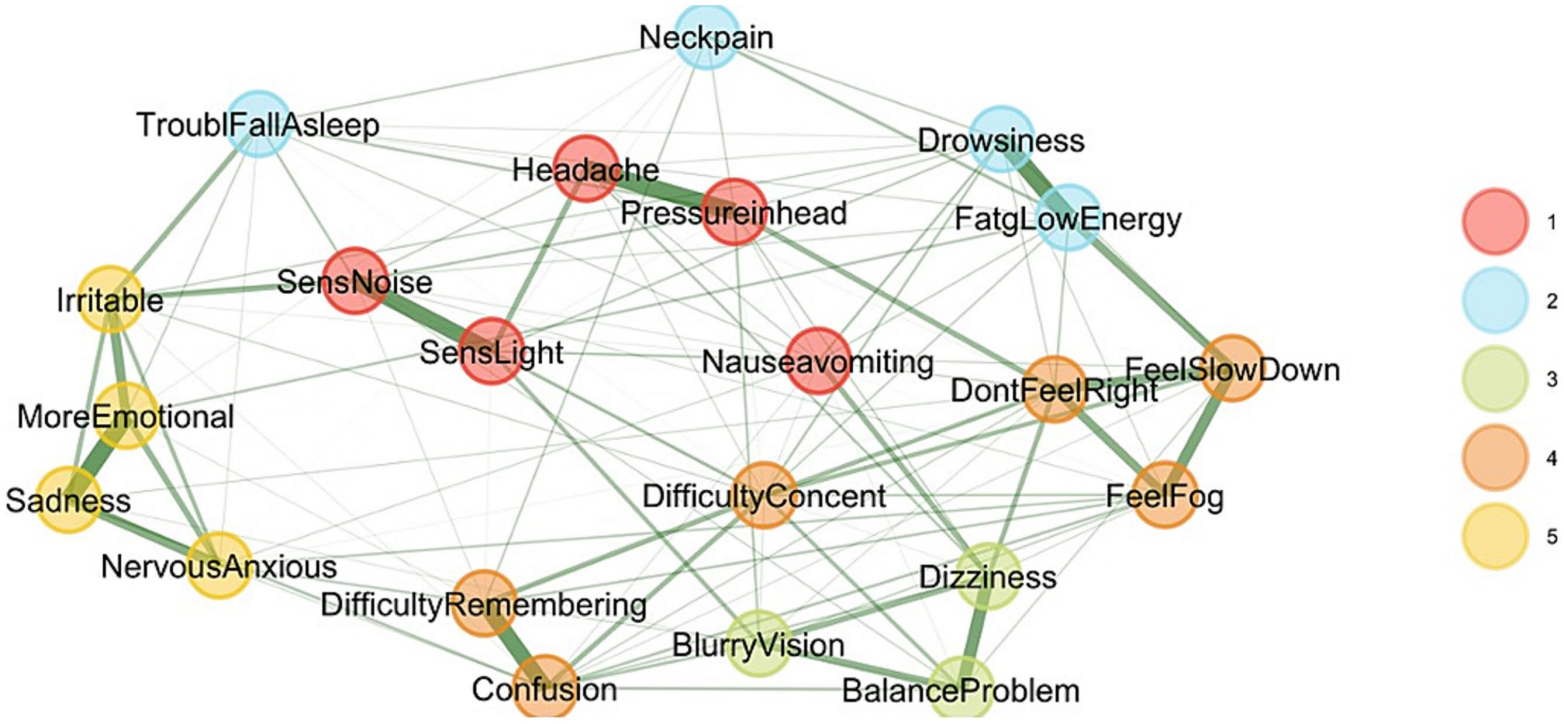
Exploratory graph analysis. This graph illustrates the SCAT3 Symptom Checklist, revealing five correlated factors. Each node represents a symptom, with lines indicating relationships between symptoms based on the stages the instrument intended to measure.

The EGA network visualization and corresponding network loadings represent different symptoms or states grouped into clusters based on their underlying correlations (Figure 1). The analysis identified five clusters. However, one factor contained only three items, and two had relatively low loadings, minimizing the variance. A total of five clusters were identified, which served as justification for further analysis using PCA and EFA to achieve a more parsimonious factor structure, reflecting symptom reporting subspaces in the SCAT. In summary, while five clusters were identified via EGA, the instability and low variance contribution of some clusters justified the use of PCA and EFA to refine the dimensional structure and improve the construct validity of the SCAT symptom domains. This step supports downstream modeling by anchoring the latent constructs in a more stable and theoretically interpretable structure.

**Figure 2:**
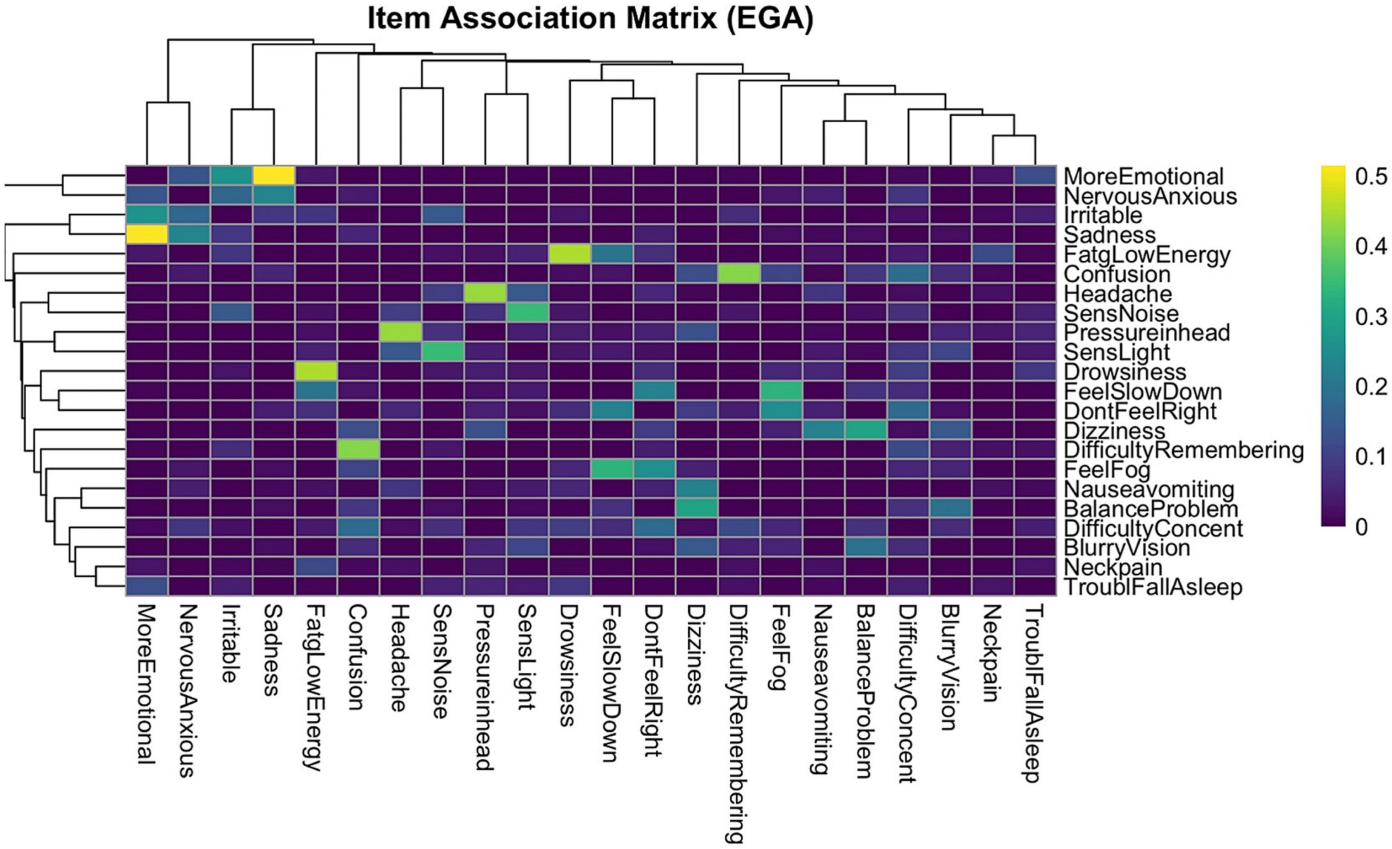
EGA item association heatmap. This dendrogram heatmap displays the strength of pairwise associations between symptoms. Items are ordered based on hierarchical clustering to visually group symptoms with similar association patterns. Color intensity reflects the magnitude of the association between symptoms, with the brighter colors indicating stronger correlations. Distinct diagonal blocks of higher association strength reflect strong within-cluster coherence, supporting the factorial structure identified by EGA. The dendrogram further reveals the nested structure of symptom groupings, offering complementary insight into the dimensional organization of post-concussion symptoms. Four distinct symptom clusters were observed; these clusters align with the four-factor structure identified through EGA and support the presence of coherent, multidimensional symptom domains in concussion presentations.

To further explore the structure of inter-item associations, a clustered heatmap was generated using the EGA-derived association matrix. Items were reordered using hierarchical clustering based on correlation distance (1–*r*), a method that prioritizes pattern similarity in item responses rather than raw magnitude (Figure 2). The resulting heatmap revealed distinct diagonal blocks of high association strength, indicating strong within-cluster coherence. In contrast, off-diagonal regions displayed weaker associations, reflecting reduced connectivity between symptom groups. The application of correlation distance was particularly appropriate in this context, as it preserved the psychological meaning of item interrelationships and supported the interpretability of the clustering solution.

#### Principal components analysis

**Figure 3:**
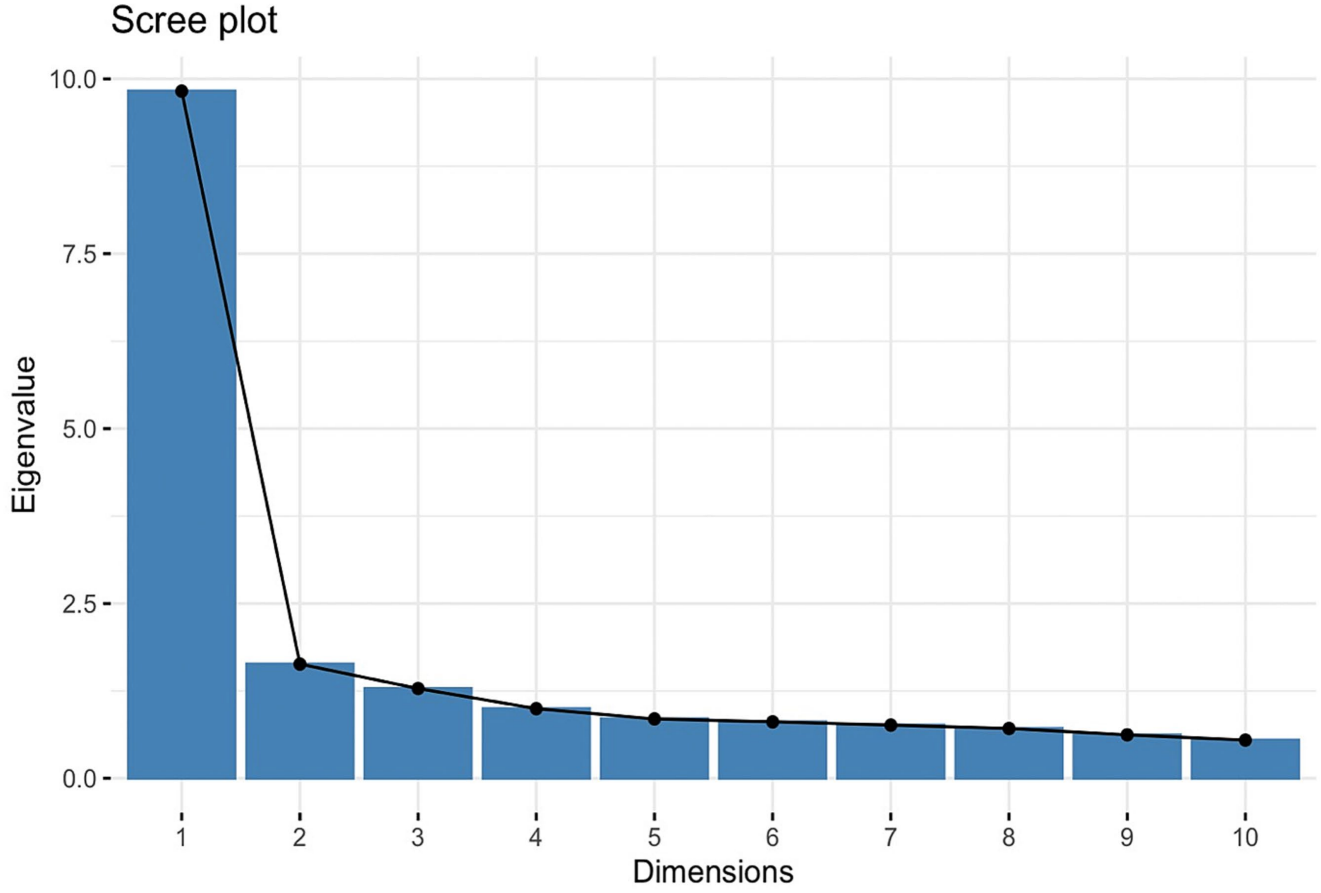
PCA scree plot. The scree plot displays eigenvalues (9.82, 1.63, 1.20, 0.998) against the dimensions, helping to identify the four factors to retain in exploratory factor analysis.

PCA identified four significant components, as rendered through a scree plot (Figure 3), which were 9.82, 1.63, 1.20, and 0.998, respectively; the fourth component’s eigenvalue nearly meets the Kaiser criterion, suggesting potential additional information. The fourth dimension explains 4.53% of the variance, leading to a higher cumulative explanation of 62.44% in the four-factor model. Including the additional fourth factor may result in a more comprehensive representation of the dataset, ensuring that subtler yet important patterns are accounted for.

#### Exploratory factor analysis (EFA)

Two models, a three-factor and a four-factor model, were compared to identify the most suitable number of factors. The four-factor model demonstrated a significantly better fit, as the three-factor model (*x*^2^(168) = 1,527.04, CFI = 0.89, TLI = 0.85, RMSEA = 0.09) demonstrated lower fit indices compared to the four-factor model to (*x^2^*(149) = 957.23, CFI = 0.94, TLI = 0.90, RMSEA = 0.07). The statistical improvements in fit support the inclusion of a fourth factor, as it better captures the multivariate subspace of the data. In turn, if the inclusion of an additional factor aligns well with known constructs, it is often best to include it. In concert with these fit values, the PCA results indicating a fourth eigenvalue close to unity suggest a four-factor model will result in a more parsimonious model.

#### Latent factor names

Based on the factor analysis shown in Table 4, the latent factors labeled as Neurocognitive, Neurophysiological, Neurosensory, and Neuropsychiatric were selected to encompass various dimensions of post-concussion symptoms. Each factor aggregates specific symptoms based on their underlying relationships and shared characteristics, providing a comprehensive understanding of the multifaceted impacts of concussions. The prefix “Neuro-” in each factor name effectively underscores the neurological basis of the symptoms associated with post-concussion syndrome:

1. *Neurocognitive*: Cognitive impairments, such as difficulty concentrating, memory problems, and mental fog, typically manifest from neurological disruption following a concussion. Labeling this factor as “neurocognitive” highlights the brain-based origin of these dysfunctions.
2. *Neurophysiological*: This factor includes symptoms that, while physical (e.g., headaches, sensitivity to light and noise), are a direct result of neurological damage resulting from concussion. This emphasizes that these symptoms are linked to neurological processes related to SRC, not just physical ailments involving other parts of the body.
3. *Neurosensory*: Symptoms grouped under this factor (e.g., blurry vision, balance problems, dizziness) are sensory-related and tied to the sensory pathways in the brain affected by the concussion. “Neurosensory” underscores the neurological origin of these sensory disturbances.
4. *Neuropsychiatric*: Emotional and behavioral symptoms (e.g., irritability, sadness, anxiety) often have neurological underpinnings. Labeling them as “neuropsychiatric” acknowledges that these symptoms are psychiatric potentially resulting from their SRC.

**Table 4:** Exploratory factor analysis loadings with latent variables.

SCAT Item #	Symptom	Neurophysiological	Neurocognitive	Neurosensory	Neuropsychological
1	Headache	0.23			
2	Pressure in head	0.51			
4	Nausea/vomiting	0.61			
8	Sensitivity to light	0.62			
9	Sensitivity to noise	0.75			
3	Neck pain	0.79			
10	Feel slowed down	0.87			
11	Feel in a fog		0.27		
12	Do not feel right		0.56		
17	Drowsiness		0.51		
13	Difficulty concentrating		0.82		
15	Fatigue/low energy		0.72		
5	Dizzy			0.42	
6	Blurry vision			0.45	
7	Balance problem			0.47	
14	Diff remembering			0.52	
16	Confusion			0.50	
18	Trouble falling asleep			0.25	
19	More emotional				0.84
20	Irritable				0.61
21	Sadness				0.83
22	Anxious				0.62

This table shows the factor loadings for different symptoms, categorized under Neurocognitive, Neurophysiological, Neurosensory, and Neuropsychological domains.

Thus, each factor aggregates specific symptoms based on their underlying relationships and shared characteristics with respect to self-reported ratings. Importantly, males and females tend to load onto these factors differently which prompts further examination into those items which may be driving these differences.

#### Linear discriminant analysis (LDA)

LDA was conducted to identify items which, in a weighted linear combination, maximized differences in how groups responded and to identify potential item biases in advance of conducting a more item-specific DIF analysis (see below). The LDA revealed significant differences in symptom reporting between males and females (Wilk’s Λ = 0.82, χ^2^ (22) = 130.56, *p* < 0.001), with an accuracy of 91% in distinguishing between the two groups. Thus, the differences between the groups are statistically significant and that the discriminant function is capturing meaningful distinctions between the genders, even though the effect size might be modest. Therefore, symptoms such as emotional distress, pressure in the head, sensitivity to noise, drowsiness, and sadness contributed most to these differences, with females reporting having these symptoms more frequently (Table 5).

**Table 5:** Linear discriminant analysis eigenvector coefficients.

Symptom	*w*	Symptom	*w*
**More emotional**	**−0.64**	Nausea	0.02
**Pressure in head**	**−0.41**	Sensitivity to light	0.07
**Sensitivity to noise**	**−0.41**	Trouble falling asleep	0.09
**Drowsiness**	**−0.30**	Difficulty remembering	0.15
**Difficulty concentrating**	**−0.28**	Balance problem	0.17
**Sadness**	**−0.26**	Blurry vision	0.18
Headache	−0.18	**Feel in a fog**	**0.26**
Dizzy	−0.11	**Anxious**	**0.26**
Fatigue	−0.05	**Do not feel right**	**0.28**
Feel slowed down	−0.04	**Irritable**	**0.30**
Neck pain	−0.01	**Confusion**	**0.45**

This table provides the eigenvector (w) coefficients for various symptoms in the linear discriminant analysis, illustrating their contribution to discriminating between groups. The discriminating coefficients > |0.25| are highlighted in bold.

**Figure 4:**
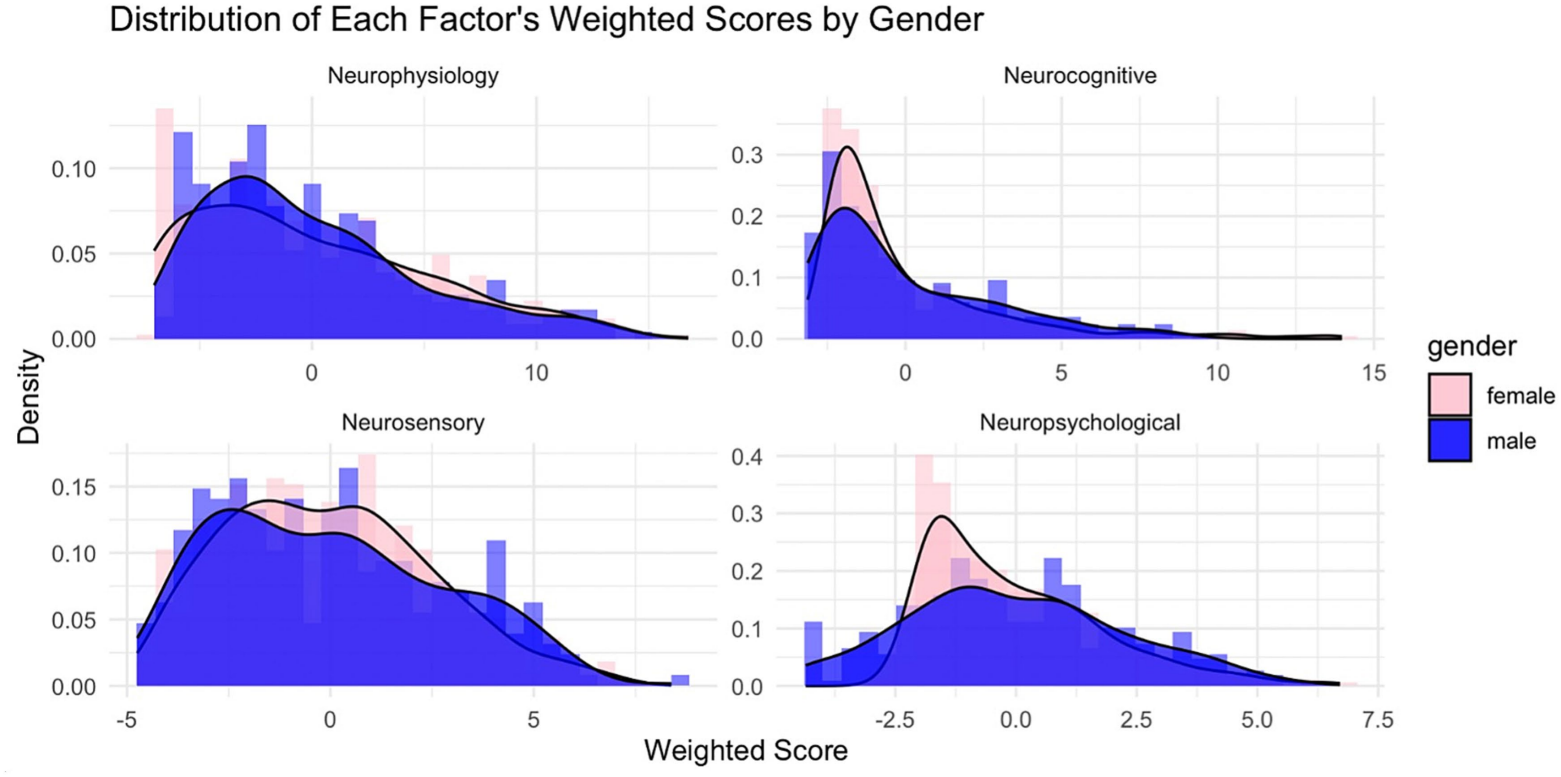
Factor score distributions by factor by gender. Factor scores were computed on each of the, so-named, *Neurophysiology*, *Neurocognitive*, *Neurosensory*, and *Neuropsychological* factors using the extracted weightings as presented in Table 3. Males (blue) and females (light red) are distinctly shown.

This density plot in Figure 4 of the LDA-derived distributions illustrates the separation of optimized symptom scores between male and female NCAA athletes. The overlap indicates some shared symptom presentation across genders. Still, the shift in the peak density for males compared to females suggests that females report specific symptoms at a slightly higher discriminant score, reflecting potential differences in symptom severity or reporting behavior between the two groups. Thus, the discriminant function holds practical importance in differentiating males from females in terms of symptom reporting.

#### Rasch partial credit model (PCM)

**Figure 5:**
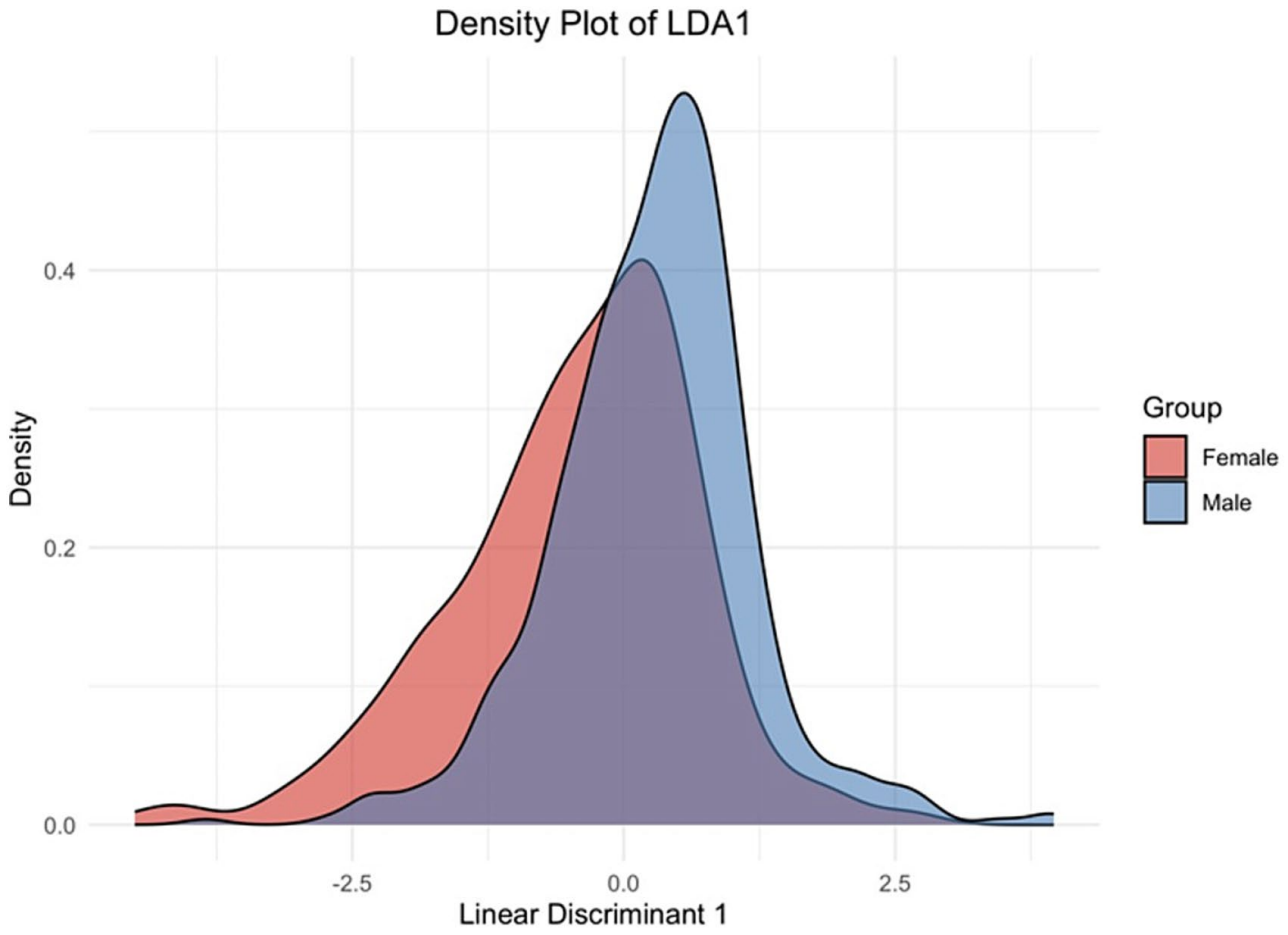
Linear discriminant density plot. The graph shows how the linear discriminant function distinguishes between the male and female groups based on the underlying symptoms or features included in the model. The *peaks* of the curves indicate the most common LDA1 scores for each gender, while the *spread* of the curves reflects the variability within each group.

This Rasch model analysis utilizing the Partial Credit Model (PCM) was conducted through WINSTEPS Version 5.8.5 (52) to examine the item thresholds for various symptoms related to a specified condition. The analysis delineated at which points along the latent trait continuum individuals were more likely to endorse successive response categories for each symptom, thus providing insight into the differential sensitivity of items as the underlying condition intensifies (45, 46) (see Figure 5).

The results in Table 6 indicated that the “Headache” has step difficulties ranging from −3.89 to 1.98, suggesting a wide range where this symptom progressively becomes more likely to be reported as the latent trait level increases from very low to high. For “Pressure in Head,” step difficulties spanned from −2.8 to 2.26, demonstrating that this symptom is relevant across a broad spectrum of the latent trait severity, with both genders starting to report this symptom at moderately low levels of the underlying trait. However, a few items displayed non-monotonic step difficulties patterns, where athletes report specific symptoms more efficiently at low severity levels but underreport them at moderate or high severity levels. Symptoms like “Nausea/Vomiting,” “Irritability,” “Sadness,” “Neck Pain,” and “Balance Problems,” respectively, suggest varied levels of the trait.

**Table 6:** Partial credit model estimated parameters and factor loadings.

		PCM estimated parameters							Factor loadings				
Factor	Item	β	δ_1_	δ_2_	δ_3_	δ_4_	δ_5_	δ_6_	1	2	3	4	H^2^
Neurophysiology^a^													
1	Headache	−7.89	−3.89	−2.76	−1.4	−0.7	0.53	1.98	0.82				0.35
2	Pressure in head	−5.20	−2.8	−1.96	−1.2	−0.31	0.69	2.26	0.72				0.36
4	Nausea/vomiting	2.77	0.19	−0.42	0.15	0.67	1.7	2.9	0.27				0.70
8	Sensitivity to light	−0.97	−1.25	−0.87	−0.27	0.24	0.91	1.61	0.56				0.47
9	Sensitivity to noise	1.18	−0.57	−0.39	0.02	0.38	1.17	3.39	0.51				0.55
Neurocognitive^b^													
3	Neck pain	1.53	−0.02	−0.71	−0.21	0.6	1.5	2.2		0.23			0.83
10	Feel slowed down	−2.39	−1.68	−1.09	−0.89	0.11	0.83	1.98		0.79			0.25
11	Feel in a fog	−1.71	−1.27	−1.14	−0.55	0.24	0.72	1.72		0.62			0.34
12	DO not feel right	−4.20	−2.43	−1.28	−1.09	−0.23	0.6	1.38		0.75			0.27
17	Drowsiness	−0.56	−0.95	−0.56	−0.41	0.12	0.87	2.2		0.61			0.35
13	Difficulty concentrating	−1.62	−1.28	−0.95	−0.63	−0.19	1.1	1.96		0.51			0.33
15	Fatigue/low energy	−2.01	−1.18	−0.91	−0.91	−0.11	0.81	1.72		0.87			0.25
Neurosensory^c^													
5	Dizzy	−3.74	−1.94	−1.35	−0.78	−0.44	0.45	1.93			0.47		0.42
6	Blurry vision	−0.42	−0.48	−0.66	−0.33	−0.11	0.77	2.37			0.52		0.50
7	Balance problem	0.10	−0.66	−0.7	−0.49	0.05	1.63	1.59			0.50		0.51
14	Diff remembering	−1.89	−0.94	−0.77	−0.64	−0.16	0.47	0.9			0.42		0.49
16	Confusion	−0.46	−0.48	−0.86	−0.55	0.4	0.87	0.95			0.45		0.41
18	Trouble falling asleep	−1.07	0.61	−1.07	−0.55	−0.72	0.45	1.26			0.25		0.76
Neuropsychological^d^													
19	More emotional	−2.30	−0.98	−1.11	−0.89	0.03	0.38	1.6				0.84	0.33
20	Irritable	−2.69	−1.37	−1.23	−0.79	−0.11	0.56	1.53				0.61	0.49
21	Sadness	−0.58	−0.58	−0.65	−0.65	0.6	0.59	0.66				0.83	0.38
22	Anxious	0.13	−0.71	−0.86	−0.6	0.36	1.48	2.78				0.62	0.56

a log Likelihood = 9,070.56.

b log Likelihood = 13,920.87.

c log Likelihood = 8,953.20.

d −2 log Likelihood = 3,731.77.

β = location parameter. δ_1_-δ_6_ = intersection parameters. H2 = communality, indicating the variance accounted for by the factors.

In examining monotonicity, most items indicated that as severity levels increase, the frequency of reporting symptoms as severe decreases. However, several symptoms display varying step difficulties, meaning reporting symptoms as a 1–2 or 5–6 symptoms may be more easily endorsed; however, reporting symptoms as moderate may be more difficult. For example, the symptom “Nausea/Vomiting” shows distorted thresholds as reporting symptoms as a 1 (δ_1_ = 0.19) or a 3 (δ_1_ = 0.15) and was more difficult to endorse than symptoms as 2 (δ_1_ = −0.42). As a result, certain symptoms may exhibit nonlinear characteristics. This means the relationship between these symptoms and their underlying causes does not follow a straightforward, predictable pattern.

#### Differential item functioning (DIF)

The results highlight significant gender differences in the reporting of concussion symptoms, with notable findings in several key symptom dimensions. DIF analysis reveals that specific symptom dimensions—such as neuropsychological, neurosensory, and neurocognitive—exhibit the most substantial gender-based differences in symptom reporting.

Table 7 shows that nine symptoms in three dimensions exhibited significant DIF. Symptoms such as “Feel in a Fog,” “Do not Feel Right,” “Fatigue,” “Dizzy,” “Balance Problem,” and “Anxious” exhibit positive levels of DIF, with female athletes consistently having reported lower symptom severity These findings support the PCM findings, as symptoms such as “Feeling in a Fog,” “Fatigue,” and “Balance Problem” indicating that males report higher severity more frequently, potentially due to underreporting at lower levels. In contrast, other symptoms such as “Difficulty Concentrating,” “More Emotional,” and “Confusion” demonstrate DIF in the opposite direction, indicating that male athletes are significantly less likely to endorse these symptoms at higher severity levels compared to females.

**Table 7:** Differential item functioning analysis parameters.

*N* = 1,021			Females = *n* = 379							Males = *n* = 642							
Item		𝛃_**F**_	δ_1_	*δ*_2_	δ_3_	δ_4_	δ_5_	δ_6_	𝛃_**M**_	δ_1_	δ_2_	δ_3_	δ_4_	δ_5_	δ_6_	𝛃_F-_𝛃_M_	Prob
Neurophysiology*^a^*																	
1	Headache	−1.07	−0.75	−1.04	−1.01	−1.22	0.00	−1.95	−1.02	−0.89	−1.14	−0.96	−0.93	−1.24	−2.96	−0.06	0.51
2	Pressure in head	−0.61	−0.74	−0.29	−0.63	−0.70	−0.57	−1.72	−0.52	−0.55	−0.32	−0.61	−0.59	−0.41	−0.27	−0.09	0.51
4	Nausea/vomiting	0.93	−0.21	0.39	0.78	0.96	1.17	1.83	0.81	−0.19	0.73	0.65	0.86	1.05	2.18	0.12	0.29
8	Sensitivity to light	0.12	−0.14	−0.14	0.24	0.15	−0.12	−0.67	0.02	−0.17	−0.02	0.12	−0.05	−0.06	−0.34	0.10	0.25
9	Sensitivity to noise	0.63	0.87	0.62	0.58	0.67	0.46	0.84	0.70	0.72	0.47	0.77	0.74	0.58	−1.06	−0.07	0.25
Neurocognitive*^b^*																	
3	Neck pain	0.75	−0.75	−1.04	−1.01	−1.22	−1.01	−1.95	0.75	−0.53	−0.27	0.49	0.89	1.34	2.14	0.00	0.32
10	Feel slowed down	−0.12	−0.89	−0.11	0.50	0.77	1.27	2.15	−0.12	0.10	−0.02	−0.12	−0.27	−0.53	−0.95	0.00	0.89
11	Feel in a fog	**0.20**	**0.45**	**0.16**	**−0.07**	**−0.32**	**−0.21**	**−0.68**	**0.00**	**−0.19**	**−0.05**	**−0.14**	**−0.02**	**−0.71**	**−0.65**	**0.20**	**<0.001**
12	Do not feel right	**−0.27**	**0.21**	**0.21**	**0.03**	**0.03**	**0.08**	**−0.40**	**−0.37**	**−0.05**	**−0.49**	**−0.51**	**−0.74**	**−0.95**	**−2.06**	**0.10**	**<0.001**
17	Drowsiness	−0.24	−0.13	−0.47	−0.38	−0.51	−0.99	−0.58	−0.19	0.20	0.13	0	−0.04	−0.29	0.47	−0.05	0.13
13	Difficulty concentrating	**−0.29**	**−0.20**	**−0.19**	**0.00**	**0.06**	**0.20**	**0.26**	**−0.08**	**−0.28**	**0.14**	**−0.05**	**−0.16**	**0.07**	**−1.98**	**−0.21**	**<0.001**
15	Fatigue/low energy	**0.38**	**1.26**	**0.55**	**0.25**	**0.19**	**−0.19**	**−0.04**	**0.27**	**1.16**	**0.26**	**0.03**	**−0.05**	**−0.10**	**−0.60**	**0.11**	**<0.001**
Neurosensory*^c^*																	
5	Dizzy	**−0.04**	**−0.36**	**−0.23**	**−0.45**	**−0.48**	**−0.40**	**−0.74**	**−0.17**	**−0.43**	**−0.32**	**−0.27**	**−0.31**	**−0.38**	**−0.56**	**0.12**	**<0.001**
6	Blurry vision	−0.28	0.12	−0.13	−0.06	−0.16	−0.23	−0.64	−0.23	−0.10	−0.48	−0.08	−0.25	−0.19	−0.46	−0.05	0.11
7	Balance problem	**0.37**	**−0.60**	**0.55**	**0.47**	**0.21**	**0.26**	**−0.24**	**0.29**	**−0.03**	**0.83**	**0.23**	**0.23**	**0.15**	**0.17**	**0.08**	**0.05**
14	Diff remembering	0.29	0.94	0.16	0.43	0.12	0.18	−0.04	0.32	0.84	0.40	0.24	0.29	−0.03	0.05	−0.02	0.28
16	Confusion	**−0.56**	**0.31**	**0.33**	**0.03**	**0.17**	**−0.26**	**−1.88**	**−0.43**	**0.16**	**0.50**	**0.28**	**0.21**	**0.49**	**−0.79**	**−0.13**	**<0.001**
18	Trouble falling asleep	−0.13	−0.25	−0.67	−0.39	0.08	0.23	0.90	−0.13	−0.79	−0.38	−0.18	0.06	0.36	0.86	0.00	0.81
Neuropsychological*^d^*																	
19	More emotional	**−0.35**	**−0.24**	**−0.46**	**−0.19**	**−0.69**	–	**−1.03**	**0.00**	**0.51**	**−0.05**	**−0.08**	**−0.03**	**−0.63**	**−0.51**	**−0.34**	**<0.001**
20	Irritable	−0.14	−0.18	−0.10	−0.17	0.00	–	−1.08	−0.32	−0.45	−0.29	−0.20	−0.68	0.40	1.78	0.18	0.08
21	Sadness	−0.07	0.26	−0.01	−0.18	−0.12	–	0.71	0.05	0.20	−0.05	0.25	−0.04	−0.20	0.25	−0.12	0.33
22	Anxious	**0.59**	**0.24**	**0.70**	**0.57**	**0.80**	–	**1.24**	**0.26**	**0.09**	**0.36**	**−0.03**	**0.72**	**0.30**	**−1.10**	**0.33**	**0.02**

β_i_ = location difficulty parameter. δ_1_-δ_6_ = intersection parameters. β_F_-β_m_ = Item Location Difference between genders. Females did not rate any of the neuropsychological symptoms as a 5 or 6, therefore, the intersection parameters were computed. Entries in bold font reflect statistically significant values.

These results, in addition to those of the LDA, provide robust evidence for gender differences in concussion symptom reporting, particularly in how male and female athletes respond to varying levels of symptom severity. Moreover, males’ symptom profile post-concussion aligns within cognitive and sensory domains, which might not affect their overall perception of symptom burden as much as the emotional and physical symptoms reported by females. Furthermore, the inflation in total symptom scores for females could be indicative of a reporting pattern where more diffuse symptoms contribute to a perception of greater severity, potentially leading to an overestimation of symptom burden.

## Discussion

The results of this study have several specific implications for the diagnosis, management, and treatment of female athletes with SRC. Firstly, the findings from this examination underscore the need for gender-sensitive approaches to concussion assessment. The SCAT self-report items, while widely used, may not be sufficient to capture the full spectrum of symptoms experienced by female athletes when used in its unidimensional form. Clinicians may need to account for the higher likelihood of emotional and sensory symptoms in females, which could contribute to a higher total symptom score but may not necessarily reflect more severe neurological impairment. Future revisions of the SCAT self-report questions and other concussion assessment tools should consider including gender-specific norms or symptom weightings to improve diagnostic accuracy and provide a more comprehensive assessment with respect to concussion symptoms experienced by women (53).

Secondly, the results suggest that female athletes may require more individualized post-concussion considerations. The presence of emotional symptoms, such as anxiety and sadness, reported more frequently by female athletes, emphasizes the importance of providing comprehensive mental health support as part of concussion recovery. Additional psychological counseling, monitoring for depression and anxiety, and ensuring that emotional symptoms likely need particular attention and best not overlooked during clinical evaluations in female athletes (13).

Third, one potential limitation of the current analysis is the unequal distribution of male and female participants, which introduces statistical and interpretive challenges when examining gender differences. Uneven sample sizes can affect the precision of parameter estimates, reduce statistical power in the smaller group, and increase the likelihood of Type II error (50). In multigroup modeling, disparities in group sizes can also inflate fit indices in favor of the larger group, potentially obscuring meaningful effects in the underrepresented group. Moreover, imbalanced samples may raise concerns about generalizability and representation, especially in studies aiming to identify sex-based disparities in concussion outcomes. To mitigate these concerns, measurement invariance testing was conducted, and sensitivity analyses were run on sport-matched subsamples (e.g., soccer, lacrosse) to reduce sport-specific variability. These additional steps helped ensure that the observed gender differences were not solely attributable to sample size disparities or contextual differences in sport exposure.

However, the analyses performed here on the SCAT’s self-reporting portion reveal that this portion of the SCAT – the most consistent component over its history—encompasses a more nuanced set of symptom sub-scales within its broader collection of symptom ratings, highlighting complexities not captured by its single aggregated score. Certain symptom clusters—such as those related to mood, migraine-like pain, and cognitive fogginess—tend to emerge as distinct factors within the overall self-reported symptomatology, suggesting that a multivariate understanding could better capture the multidimensional experience of concussion. Additionally, male and female athletes often report differing symptom severities on specific items, with female athletes generally rating symptoms like headache, nausea, and emotional sensitivity more intensely than their male counterparts. These differences point to a need for gender-specific symptom profiling within the SCAT’s self-report section to capture the variations in how concussions manifest and are experienced across athletes. Recognizing and addressing these sub-scales and gender-related response differences could make the SCAT a more finely tuned tool for assessing and monitoring concussion, leading to more individualized and effective clinical management.

Multivariate analyses revealed the presence of four distinct symptom clusters, reinforcing the argument for a multidimensional approach to concussion symptom assessment. Prior studies support that an effective concussion care protocol must acknowledge the neurocognitive, neurophysiological, neurosensory, and neuropsychiatric dimensions of symptoms (4, 34). The unidimensional model employed by SCAT3, as analyzed here, is likely insufficient to capture the intricate interplay among these dimensions, potentially leading to misdiagnoses or mismanagement in athletic settings.

### Gender differences in SRC self-reporting

The present investigation highlights significant gender disparities in post-concussion symptom reporting among a large sample of NCAA student-athletes obtained, underscoring the limitations of the SCAT3 Symptom Severity Checklist’s traditionally scored unidimensional structure. Female athletes demonstrated a higher overall symptom severity, particularly within the emotional and sensory domains, suggesting an inherent bias in symptom assessment that warrants further clinical attention. These findings are crucial to understanding the implications of personalized concussion management strategies, especially given the historical tendency to underestimate the severity of symptoms in male athletes.

Additionally, it has been suggested that gender differences in symptom reporting may stem from cultural influences, with male athletes often underreporting symptoms due to societal pressures to exhibit “toughness” in competitive environments (71). Such behavioral discrepancies can compromise the accuracy of symptom assessment and contribute to inflated severity scores for female athletes within the critical period following injury. This calls for a reevaluation of current assessment tools, as failing to account for these gender-based differences may result in male athletes being inaccurately perceived as less symptomatic, thereby jeopardizing their health and recovery trajectory.

The evidence emphasizes the need to modify existing concussion assessment methods, particularly the SCAT3. Clinicians must adopt a nuanced understanding of symptom reporting that integrates gender-specific considerations to enhance the accuracy of diagnoses, inform appropriate management protocols, and ultimately improve outcomes for all athletes.

### Limitations of the present investigation

This investigation was conducted on NCAA athletes, and it is important to note that these results cannot be generalized to youth, high school, or professional sports. Most of the participants in the CARE Consortium sample attended academic institutions with well-funded athletic programs, which may reflect a greater attention to concussion symptoms, better quality of treatment, and more formalized programs for concussion management. Therefore, it is unclear if such results would be the same or similar in participants drawn from smaller athletic programs, historically Black colleges and universities (HBCUs), or other ethnically unique programs. Consequently, it would be advantageous to conduct further studies on more comprehensive, national samples including different age groups, contact vs. non-contact, youth, collegiate, and professional sports, as well as socioeconomic backgrounds to better understand and generalize the properties of SCAT as they pertain to both male and female athletes.

Indeed, this examination could not consider any psychological or social factors impeding symptom reporting. It will be important for future research to include variables that reflect how psychological and social factors influence gender biases at different stages of recovery (20, 31, 54, 55, 72). Researchers may want to expand upon the current Rasch Partial Credit Model to account for additional parameters to understand better how much external factors influence accurate symptom reporting. Rasch modeling will be a useful tool for researchers in the concussion field to evaluate the relationship between sociological pressures, such as reporting intentions, and diagnostic measures, such as symptom presentation, on the variability of recovery length.

### Future SCAT assessment recommendations

A female-athlete-specific section in future versions of the SCAT is essential due to accumulating evidence that female athletes experience and report concussion symptoms differently from their male counterparts. Female athletes are more likely to report symptoms such as migraines, mood disturbances, and neck pain after a concussion, which may be linked to anatomical, hormonal, and physiological differences. These differences not only affect symptom severity but can also influence recovery duration, as female athletes often report prolonged symptom durations compared to male athletes.

The current SCAT assessment, however, remains largely agnostic to gender differences, potentially leading to under-recognition or misinterpretation of symptoms in female athletes. By incorporating a new section in future versions of the SCAT dedicated to symptoms and issues more commonly reported by female athletes, such as hormonal influences on mood and menstrual cycle irregularities, the SCAT could provide a more accurate and comprehensive picture of concussion specifically as it pertains to women. This tailored approach would support clinicians in identifying concussion effects more precisely and creating individualized care plans that consider the unique recovery patterns of female athletes. A female-athlete-specific section in the SCAT would, thus, represent a critical step forward in equitable, evidence-based concussion care for athletes across all sports and competition levels.

Our analysis revealed that gender differences in the self-reporting of concussion symptoms, as measured by the SCAT assessment, are best conceptualized through a multidimensional structure comprising four distinct symptom subscales: neurocognitive, neurophysiological, neurosensory, and neuropsychological. This novel factor solution advances the field by offering a more nuanced framework for interpreting symptom patterns following sport-related concussion, particularly in the context of sex-specific variation. By identifying these four latent domains through combined exploratory graph analysis, principal component analysis, and linear discriminant analysis, the current study contributes to a growing body of research that emphasizes the value of domain-specific symptom modeling over traditional total score comparisons. Importantly, this work builds on—and helps reconcile—prior studies that have reported inconsistent SCAT factor structures across different populations and statistical approaches (28, 29, 34). Clinically, these findings support the development of more individualized and gender-informed approaches to post-injury symptom tracking, which may ultimately improve diagnostic precision, monitoring of recovery, and return-to-play decision-making in both male and female athletes (28, 29, 34).

More specifically, to enhance the SCAT’s sensitivity to the unique experiences of female athletes with SRC, based upon the discriminant analysis performed here, five additional assessment items concerning the following might be considered:

*Menstrual Cycle Changes and Symptoms:* Concussions can impact the menstrual cycle resulting in irregularities or heightened premenstrual symptoms, which, themselves, may complicate recovery. An item asking about recent changes in menstrual patterns or cycle-related symptom severity would allow clinicians to monitor potential hormonal impacts that may influence both symptoms and healing time in female athletes.
*Mood Changes and Emotional Sensitivity:* Research indicates that female athletes are more likely to report mood swings, irritability, and emotional sensitivity post-concussion. Adding an item specifically assessing mood disturbances (“Have you experienced increased mood swings, irritability, or emotional sensitivity?”) could help clinicians monitor this common symptom and inform recovery strategies.
*Sleep Disturbances Related to Hormonal Fluctuations:* Likewise, hormonal fluctuations can affect sleep quality in female athletes, which may exacerbate concussion recovery. A targeted item assessing sleep issues with attention to any recent menstrual or hormonal changes (e.g., “Have you experienced disrupted sleep, especially during your menstrual cycle?”) could give a fuller picture of factors influencing recovery.
*Migraine-Like Symptoms:* While headache is included in the SCAT, female athletes often report migraine-like symptoms, such as throbbing pain and heightened sensitivity to light or sound, more frequently than male athletes following concussion. A more specific item assessing the nature and intensity of headache symptoms (e.g., “Is the headache migraine-like, with throbbing or sensitivity to light/sound?”) could capture this experience more accurately.
*Neck Pain and Whiplash Sensitivity:* Female athletes suffer higher instances of neck pain and whiplash-like symptoms post-injury, likely due to anatomical and muscular differences (56). A new item assessing neck-related symptoms or pain more carefully would allow athletic trainers and clinicians to differentiate these from primary concussion symptoms, thereby refining diagnosis and treatment options.

Incorporating a new section including items on these topics could make future versions of the SCAT more responsive to the unique symptom experiences of female athletes, potentially leading to more personalized and effective concussion management strategies.

## Conclusion

In conclusion, this quantitatively-focused examination highlights the multidimensional nature of concussion self-reporting of symptoms as measured by the SCAT Symptom Severity Checklist and underscores the importance of (1) the multi-factorial nature of the SCAT symptom self-reporting, as well as (2) more carefully considering gender differences in concussion assessment. The findings suggest that an athlete-gender-agnostic approach to concussion symptom severity may not be appropriate and that gender-specific considerations should be integrated into clinical assessments and treatment plans. By adopting a more nuanced, multidimensional approach, athletic training staff and healthcare providers can ensure more precise diagnosis and tailored interventions, ultimately improving outcomes for all athletes. Neuropsychological testing is recommended to remain a key component in evaluating complex concussions, although it is not currently considered essential for assessing simple concussions. While the SCAT assessment and the analyses examined here significantly enhances both the understanding of concussion effects and the potential for management of individual athletes, they should not serve as the sole basis for decisions regarding time away from play or return-to-play readiness. Nevertheless, the clinical management of concussions specific to the symptoms female athletes tend to report is not often part of current SRC assessments (57) as currently practiced, as exemplified by the SCAT. If included in future versions of the SCAT, the additions of self-report items, as recommended here, strategically added to the sub-scale structure or as its own sub-scale, altogether, would undoubtedly provide important context to individualized SRC treatment approaches.

## Data Availability

Publicly available datasets were analyzed in this study. This data can be found at: https://fitbir.nih.gov.
